# Full shut-off of *Escherichia coli* RNA-polymerase by T7 phage requires a small phage-encoded DNA-binding protein

**DOI:** 10.1093/nar/gkx370

**Published:** 2017-05-09

**Authors:** Aline Tabib-Salazar, Bing Liu, Andrey Shadrin, Lynn Burchell, Zhexin Wang, Zhihao Wang, Moran G. Goren, Ido Yosef, Udi Qimron, Konstantin Severinov, Steve J. Matthews, Sivaramesh Wigneshweraraj

**Affiliations:** 1 MRC Centre for Molecular Microbiology and Infection, Imperial College London, London SW7 2AZ, UK; 2 G.K. Skryabin Institute of Biochemistry and Physiology of Microorganisms, Russian Academy of Sciences, Pushchino, Moscow 142290, Russia; 3 Department of Clinical Microbiology and Immunology, Sackler School of Medicine, Tel Aviv University, Tel Aviv 69978, Israel; 4 Waksman Institute of Microbiology, Rutgers, The State University of New Jersey, 190 Frelinghuysen Road, Piscataway, NJ 08854-8020, USA

## Abstract

Infection of *Escherichia coli* by the T7 phage leads to rapid and selective inhibition of the bacterial RNA polymerase (RNAP) by the 7 kDa T7 protein Gp2. We describe the identification and functional and structural characterisation of a novel 7 kDa T7 protein, Gp5.7, which adopts a winged helix-turn-helix-like structure and specifically represses transcription initiation from host RNAP-dependent promoters on the phage genome via a mechanism that involves interaction with DNA and the bacterial RNAP. Whereas Gp2 is indispensable for T7 growth in *E. coli*, we show that Gp5.7 is required for optimal infection outcome. Our findings provide novel insights into how phages fine-tune the activity of the host transcription machinery to ensure both successful and efficient phage progeny development.

## INTRODUCTION

Many bacteriophages (phages) use diverse mechanisms to repurpose, redirect or inhibit the bacterial transcription machinery, the RNA polymerase (RNAP), to coordinate phage gene expression and developmental needs during infection (1,2). T7 is a widely studied obligate lytic phage of *Escherichia coli* (*Ec*). The 56 gene products (Gps) of T7 are categorised as early (class I: Gp0.3-1.3), middle (class II: Gp1.6-6.3) and late (class III: Gp6.5-19.5) to reflect the timing of their expression during the infection process (3,4). Early and middle genes generally encode proteins required for phage RNA synthesis, DNA replication and host takeover; the late genes specify T7 virion assembly and structural proteins. The *Ec* RNAP containing the major housekeeping σ^70^ factor initially catalyses the entry of the T7 DNA into the cell by transcribing the early genes from three strong early gene promoters *T7 A1, A2* and *A3* (5). The host RNAP is then shut off by the coordinated action of the early gene product Gp0.7 and the essential middle gene product Gp2; the viral single-subunit RNAP (T7 RNAP, Gp1, a product of an early gene) transcribes the middle and late viral genes. The shutting down of host RNAP is crucial for the successful completion of the infection cycle: Gp0.7 is a protein kinase that phosphorylates the *Ec* RNAP, leading to increased termination of transcription at sites located between the early and middle genes on the T7 genome (6); and Gp2 binds in the main DNA binding channel in *Ec* RNAP and thereby prevents the formation of the transcriptionally proficient open promoter complex (RPo) at the *T7 A1-3* promoters (7). Gp0.7 is dispensable for T7 growth under standard laboratory conditions, but becomes essential when Gp2 function is compromised (6). However, Gp2 is indispensable for T7 growth and its absence leads to interference by the host RNAP transcription initiated from *T7 A1-3* promoters, with middle and late T7 gene transcription by the T7 RNAP (8).

Although several early and middle Gps of T7 have been assigned biological roles in the T7 infection cycle (9), many remain uncharacterised. One of these is Gp5.7, a 7 kDa protein of unknown function. Gene *5.7* is located downstream of gene *5.5* (encodes the *Ec* HNS inhibitor; see later) and the two genes overlap by one nucleotide (4), suggesting that a fusion *gene 5.5/5.7* product resulting from a frame-shift translation could exist. However, Zhang *et al.* found that a *gene 5.5/5.7* fusion protein is not expressed (10). We demonstrate that Gp5.7 is an *Ec* RNAP-binding protein, which specifically represses the ability of the host RNAP to initiate transcription from the early *T7 A1, A2* and *A3* promoters. We have elucidated the solution structure of Gp5.7, which reveals a fold similar to the winged helix-turn-helix DNA binding motif and provides a mechanistic insight into how Gp5.7 specifically alters *Ec* RNAP performance at *T7 A1-A3* promoters. A T7 mutant that lacks Gp5.7 is compromised for growth in *Ec*, which underscores the involvement of Gp5.7 in the optimal inhibition of host RNAP and thus T7 growth in *Ec*.

## MATERIALS AND METHODS

### Gp5.7 expression and purification

T7 *gene 5.7* was PCR amplified from T7 DNA using primers AS1 and AS2 (Supplementary Table S3) containing restriction sites for NdeI and BamHI, respectively, and cloned into the same sites in plasmid pET33b+ (Novagen) to create pSW33b::*gp5.7*. This plasmid was then digested with XbaI and HindIII restriction endonucleases and the resulting fragment containing T7 *gene 5.7* was cloned into the same sites in pBAD33 (ATCC) to create pBAD18::*gp5.7*, which expresses Gp5.7 as a fusion protein containing a six-histidine (6xHis) and heart muscle kinase tags at its N terminus. All Gp5.7 mutants were created by site-directed PCR mutagenesis using pBAD18::*gp5.7* and appropriate primers (available upon request) as the template and confirmed by DNA sequencing. For the biochemical experiments, recombinant wild-type (WT) and mutant Gp5.7 was made by nickel affinity purification from *Ec* strain MC1061. Briefly, the culture of MC1061 cells containing pBAD18::*gp5.7* was grown at 37°C to an OD_600_ of ∼0.4 and Gp5.7 expression was induced with 0.04% (w/v) arabinose. The cells were left to continue growing at 37°C for 4 h before harvesting. The cell pellet was re-suspended in Binding buffer (25 mM NaH_2_PO_4_ pH 7.0, 0.5 M NaCl, and 5% (v/v) glycerol) containing cocktail of protease inhibitors and lysed by sonication. The cleared cell lysate was loaded onto a His-Trap HP column [GE Healthcare Life Sciences], which was connected to a fast protein liquid chromatograph machine. The purified protein was eluted over a 40 ml gradient of 0–100% Elution buffer (Binding buffer + 1 M imidazole pH 7.0) according to manufacturer's instructions. The purified protein was dialysed into Storage buffer (10 mM Tris–HCl pH 6.8, 50 mM NaCl, 0.1 mM EDTA, 20% (v/v) glycerol, 1 mM DTT) and stored in aliquots at –80°C. For NMR studies, *Ec* strain MC1061 containing pBAD18::*gp5.7* was grown in Silantes OD1 superrich medium labeled with ^15^N and ^13^C and Gp5.7 expression was induced with 0.04% l-arabinose when the OD_600_ of the culture reached 0.4; the cells were harvested following overnight incubation at 18°C and Gp5.7L42A was purified as described above for the WT protein. The purified protein was then treated with 50 μg/unit of enterokinase protease to remove the 6xHis tag; the cleaved protein was then isolated by re-applying the mixture onto a His-Trap HP column. The eluate was then dialysed into NMR spectroscopy buffer containing 50 mM NaH_2_PO_4_ at pH 6, 350 mM NaCl and 0.2 mM TCEP. The samples were subsequently concentrated to ∼5 mg/ml for NMR experiments.

### Pull-down assays

The MagneHis™ Protein Purification System (Promega) was used for pull-down assays: Approximately 0.02 mg of recombinant 6xHis tagged Gp5.7 in Binding buffer (20 mM Na_2_PO_4_, 50 mM NaCl, 5% glycerol, pH 7.0) was added to 100 μl resin and incubated for 30 min at 4°C. Cleared *Ec* whole-cell lysate was obtained from exponentially growing *Ec* strain MC1061, which was added to resin containing Gp5.7 and incubated for 1 h at 4°C. For pull-down assays involving recombinant proteins, the core and holoenzyme forms of the *Ec* RNAP were purchased from NEB; M0550S and M0551S, respectively and ∼0.02 mg was added to resin containing Gp5.7 and incubated for 1 h at 4°C. The beads were washed three times in 1 ml wash buffer (20 mM Na_2_PO_4_, 500 mM NaCl, 5% (v/v) glycerol, 30 mM imidazole, pH 7.0) for 10 min to remove any non-specific protein–protein interaction. To elute samples from beads, 50 μl of binding buffer and 50 μl of Laemmli 2x concentrate SDS Sample Buffer was added to beads and boiled for 15 min. Ten microliters was loaded on a 10–15% SDS-PAGE alongside either 5 μl of PageRuler™ Prestained Protein Ladder and stained with Coomassie Brilliant Blue or 5 μl of MagicMark™ XP Western Protein Standard and proteins were detected by western blotting.

### Electrophoretic mobility shift assays

Fifty nanomolar ^32^P-labeled recombinant Gp5.7 was incubated with varying amounts of *Ec* core RNAP (NEB) at 37°C for 10 min in 40 mM Tris–HCl pH 8, 10 mM MgCl_2_, 1 mM DTT and 100 mM NaCl and in the presence of 0.5 μg/μl α-lactoglobulin. The reaction was separated on a non-denaturing gel with the addition of Commassie G250 (Sigma-Aldrich) and the gel was run for 90 min and 100 V in the presence of a cooling block. The dried gel was then analysed by autoradiography.

### Isothermal titration calorimetry (ITC)

ITC experiments were performed on an ITC-200 instrument (Microcal). Gp5.7L42A and double-stranded (ds) DNA probes were prepared in the same NMR spectroscopy buffer as above. Gp5.7L42A and ds DNA probes were present at 0.2 and 3 mM, respectively, and the ds DNA probe was titrated by 20 injections of 2 μl every 100 s at 25°C. The raw data were integrated, normalized for the molar concentration and analysed using Origin7.0 according to a 1:1 binding model.

### Western blotting

The SDS-PAGE gel was transferred onto polyvinylidene difluoride (PVDF) membrane (0.45 μm) using Trans-Blot^®^ Turbo™ Transfer System [Bio-Rad] device and processed according to standard molecular biology protocols. The primary antibodies were used at the following titres: anti-*Ec* RNAP β–subunit antibody at 1:1000 [8RB13—abcam], anti-*E*c RNAP α-subunit antibody at 1:1000 and anti-6xHis tag^®^ antibody (HRP) at 1:5000 [ab1187—abcam]. The secondary antibody Rabbit Anti-Mouse IgG H&L (HRP) was used at 1:2500 [ab97046—abcam]. Bands were detected using an Amersham ECL Western Blotting Detection Reagent [GE Healthcare Life Sciences] and analysed on Chemidoc using the Image Lab Software.

### *In vitro* promoter-independent and dependent transcription assays

These were conducted and analysed exactly as previously described (7). The sequence of the promoter-less minimal scaffold template is provided in Figure 2A. The sequences of the different promoter sequences used in Figure 2B–D are listed in Supplementary Table S3. For all the *in vitro* transcription assays, the core RNAP was obtained from NEB and the *Ec* σ^70^ was purified exactly as described by Nechaev and Severinov (11).

### Bacterial growth attenuation assays

These were conducted exactly as previously described by Shadrin *et al*. (12).

### NMR structure determination and chemical shift experiments

NMR spectra were collected at 310K on Bruker DRX600 and DRX800 spectrometers equipped with cryo-probes. Spectral assignments were completed using our in-house, semi-automated assignment algorithms and standard triple-resonance assignment methodology (13) using the three-dimensional (3D) HNCA, HNCO, HN(CO)CA, HNCACB and CBCA(CO)NH. H_α_ and H_β_ assignments were obtained using HBHA (CBCACO)NH and the full side-chain assignments were extended using HCCH-total correlation (TOCSY), (H)CC(CO)NH TOCSY and ^1^H–^15^N/^13^C NOESY-HSQC experiments. The two NOESY spectra were also used to provide the distance restraints in the final structure calculation (14). The ARIA protocol was used for completion of the NOE assignment and structure calculation. The frequency window tolerance for assigning NOEs was ±0.025 and ±0.03 ppm for direct and indirect proton dimensions and ±0.6 ppm for both nitrogen and carbon dimensions. The ARIA parameters p, Tv and Nv were set to default values. One hundred eight dihedral angle restraints derived from TALOS+ (15) were also implemented. The 10 lowest energy structures had no NOE violations >0.5 Å and dihedral angle violations >5°. The structural statistics are shown in Supplementary Table S1. For the NMR chemical shift experiments, ds DNA probes (Supplementary Table S2) was added to ^15^N-labeled Gp5.7 according to stoichiometric ratio to perform NMR titration. Maximal five-fold DNA was added to Gp5.7L42A in order to broad out the entire spectra. The 10 structures were deposited to BMRB (ID: 34024) and PDB (accession number: 5LGM).

### Construction of T7 Δ*gp5.7* phage

Plasmid pUC5.7::*trxA* was constructed to replace *gene 5.7* in the T7 genome with the *Ec trxA* gene, a positive selection marker for T7 grown on hosts lacking *trxA*. The *trxA* in the pUC5.7::*trxA* plasmid was flanked by 50 bp upstream and downstream of the DNA sequence encoding *gene 5.7*. The plasmid was constructed by PCR amplification of *Ec trxA* gene by using primers MG5F and MG5R (Supplementary Table S3), which contained the NdeI and XbaI restriction sites, respectively. The PCR fragment was then digested and ligated to a compatibly digested pUC19 plasmid. T7 encoding *trxA* instead of the *gene 5.7* was constructed by homologous recombination as described previously (16) using pUC5.7::*trxA* plasmid. The obtained lysate was used to infect *Ec* lacking *trxA*. Single emerging plaques were purified and the correct insertion was verified by DNA sequencing.

### T7 phage infection and plating assays

*Ec* strain MG1655 *rpoC*-FLAG and JE1134 were grown in LB at 30°C to an OD_600_ of ∼0.5. For infection with T7 phage, 1 mM CaCl_2_ and 1.9 × 10^8^ of T7 WT and T7 Δ*gp5.7* mutant phage were added to each culture in triplicate and OD_600_ reading taken every 10 min until complete lysis was obtained or until 120 min after T7 infection. For the plating assays, *Ec* strain MG1655 *rpoC*-FLAG culture was grown to OD_600_ 0.7–0.8 at 37°C and 100 μl of the culture was mixed with 10 μl of T7 WT or T7 Δ*gp5.7* phages (∼ corresponding to 10 plaque forming units) and incubated with 1 mM CaCl_2_ for 10 min at 30°C to allow the phage to adsorb to the bacterial cells. Three milliliters of 0.7% (w/v) LB agar, pre-warmed to 50°C, was added and the mixture was overlaid on LB plates. After 15–18 h at 30°C, plaques were counted. Only plaques of diameter ≥0.2 cm were taken into account. Three separate experiments were performed, and average values were calculated for each bacterial colony infected with T7 WT or T7 Δ*gp5.7*.

## RESULTS

### Gp5.7 is an *Ec* RNAP-binding protein

**Figure 1.**
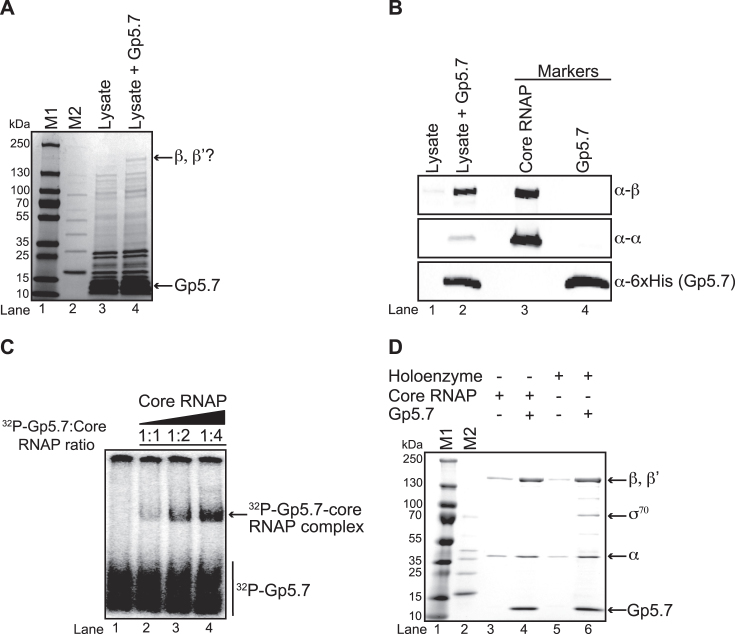
Gp5.7 is an *Ec* RNAP σ^70^ binding protein. (**A**) Image of a denaturing gel showing that Gp5.7 pulls down the RNAP from *Ec* whole cell lysate. The migration positions of the Gp5.7 and the suspected β and β’ subunits of the *Ec* RNAP are indicated. M1 is PageRuler™ Prestained Protein Ladder and M2 is MagicMark™ XP Western Protein Standard. (**B**) Image of a Western blot probed with anti-β, anti-α and anti-6xHis (to detect Gp5.7) antibodies. Lanes 1 and 2 contain the same sample as in lanes 3 and 4, respectively, of the denaturing gel in (A); lanes 3 and 4 contain the purified core RNAP and Gp5.7, respectively, as markers. (**C**) Image of a non-denaturing gel showing results of the electrophoretic mobility shift assay using 50 nM ^32^P-labeled recombinant Gp5.7 with varying amounts of *Ec* core RNAP. Lane 1 only contains ^32^P-labeled recombinant Gp5.7 whilst lanes 2 to 4 contain 50 nM ^32^P-labeled recombinant Gp5.7 with increasing amounts of *Ec* core RNAP. The migration positions of ^32^P-Gp5.7 and the ^32^P-Gp5.7-core RNAP complex are indicated. (**D**) Image of a denaturing gel showing results of a pull-down assay conducted as in (A) but using purified core (lane 4) and holoenzyme (lane 6) forms of the *Ec* RNAP. The reaction components in each lane and migration of β, β’, α and σ^70^ subunits and Gp5.7 are indicated.

In order to identify the potential interacting partner(s) of Gp5.7 in *Ec*, we made recombinant amino-terminal 6xHis tagged Gp5.7, immobilised it onto nickel-charged affinity resin and incubated the Gp5.7 containing resin with whole-cell lysates from exponentially growing *Ec* cells. After washing, Gp5.7-interacting proteins were eluted and separated by denaturing SDS polyacrylamide gel electrophoresis (SDS-PAGE). As shown in Figure 1A, inspection of the coomassie blue stained gel revealed a highly enriched doublet of bands migrating at ∼150 kDa in lysate with Gp5.7 (lane 4) compared to the control reaction (containing resin without Gp5.7; lane 3). We suspected that the doublet could be the β, β’ subunits of the *Ec* RNAP. To confirm this, we probed the denaturing gel with antibodies against the β and α subunits. Results shown in Figure 1B revealed that the antibodies against the β and α subunits cross-reacted with the bands migrating at ∼150 and ∼36 kDa, respectively, suggesting that Gp5.7 is potentially an *Ec* RNAP binding protein. To ascertain that Gp5.7 can interact with the core RNAP, we incubated a fixed amount of recombinant ^32^P-labeled Gp5.7 and varying amounts of *Ec* core RNAP and separated the reactions on a non-denaturing gel. Results shown in Figure 1C clearly demonstrates that ^32^P-labeled Gp5.7 can form a binary complex with the core RNAP in a dose-dependent manner. Next, we repeated the pull-down assays with purified recombinant *Ec* RNAP with and without σ^70^ and, following several washes, analysed the eluted samples on a denaturing gel. The results unambiguously established that Gp5.7 is indeed an *Ec* RNAP binding protein, which interacts with both σ^70^-free (core) and σ^70^-bound (holoenzyme) forms of the *Ec* RNAP (Figure 1D).

### Gp5.7 specifically represses *Ec* RNAP activity at T7 early gene promoters

**Figure 2.**
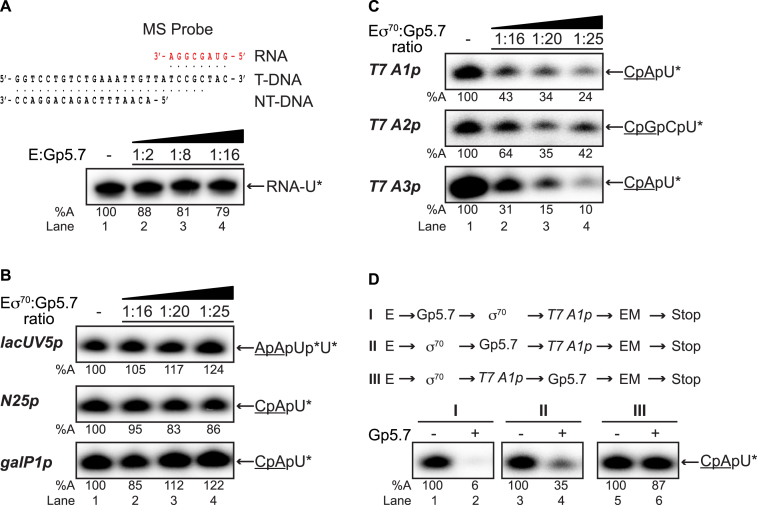
The effect of Gp5.7 on *Ec* RNAP activity. (**A**) Autoradiograph of a denaturing gel showing the ability of the core *Ec* RNAP to synthesise the RNA-U product (asterisk indicates the radiolabeled nucleotide) from the MS probe. The sequence of the MS probe is schematically shown at the top of the autoradiograph. (**B**) Autoradiograph of denaturing gels showing the ability of the *Ec* RNAP holoenzyme to synthesise a dinucleotide-primed RNA product from the *lac*UV5, N25 and *gal*P1 promoters. The dinucleotide used in the assay is underlined and the asterisks indicate the radiolabeled nucleotide(s). (**C**) As in (B) but *T7 A1, A2* and *A3* promoters were used in the reactions. (**D**) As in (B) but Gp5.7 was added to the reaction at the different stages (I-III) during transcription initiation as indicated in the reaction schematics I–III using the *T7 A1* promoter. In (A)–(D), the percentage of RNA transcript synthesised (%A) in the reactions with Gp5.7 with respect to reactions with no Gp5.7 is given at the bottom of the gel and the value obtained in at least three independent experiments fell within 3–5% of the %A value shown.

Having established that Gp5.7 is an *Ec* RNAP binding protein, we next determined how Gp5.7 affects the activity of the enzyme. To initially determine whether Gp5.7 affects the catalytic activity of *Ec* RNAP, we measured the activity of the core enzyme on a promoterless minimal nucleic acid scaffold template (hereafter called the minimal scaffold (MS) probe). The MS probe consists of an 18-nucleotide-long DNA duplex and an eight-nucleotide-long RNA–DNA heteroduplex separated by two unpaired DNA bases (Figure 2A) (17). Thus, the MS probe lacks the consensus promoter DNA sequences recognised by σ^70^. The addition of α^32^P-UTP to the E-MS probe complex results in the synthesis of a nine-nucleotide-long α^32^P-UTP-labeled RNA product, hereafter called RNA-U. Results in Figure 2A revealed that the catalytic activity of *Ec* RNAP was unaffected by the presence of Gp5.7 in the reaction. We next conducted *in vitro* multiple-round transcription initiation assays on well-characterised σ^70^-dependent promoters (*lac*UV5, N25, and *gal*P1) to determine if Gp5.7 affects the ability of the *Ec* RNAP to bind to the promoter, nucleate DNA strand-separation and synthesise a dinucleotide-primed RNA product. All the DNA probes used in the transcription initiation assays were 65 base pairs long with 20 base pairs of sequences downstream of the transcription start site at +1, except *T7 A2p* and *T7 A3p* which are 59 base pairs long with 14 base pairs of sequences downstream of the transcription start site at +1 (Supplementary Table S3). As shown in Figure 2B, when Eσ^70^ was pre-incubated with Gp5.7 prior to the addition of the promoter probe, no detectable effect in the amount of the RNA product synthesised by the *Ec* RNAP was detected irrespective of the promoter used in the assay or the amount of Gp5.7 present in the reaction. Strikingly, however, under identical conditions, Gp5.7 had a substantial specific and dose-dependent adverse effect on the ability of the *Ec* RNAP to synthesise the RNA product from the T7 early gene promoter *A1* (Figure 2C and see later Supplementary Figure S1E). Since T7 early genes are expressed from three *Ec* RNAP-dependent promoters (*T7 A1*, A2 and *A3*), we tested whether Gp5.7 also adversely affects the *Ec* RNAP on T7 early gene promoters *A2* and *A3*. The results shown in Figure 2C clearly indicated that this is indeed the case and revealed that the ability of *Ec* RNAP to synthesise a RNA product from the *T7 A2* and *A3* promoters were equally compromised in the presence of Gp5.7 as from the *T7 A1* promoter.

To determine which step during transcription initiation Gp5.7 affects on the *T7 A1* promoter, Gp5.7 was added at different points of the reaction (conditions I-III; Figure 2D). Results indicated that Gp5.7 had a detectable adverse effect on the amount of the RNA product synthesised when it was added to the RNAP before holoenzyme formation (condition I) or before the initial engagement of RNAP with the promoter to form the closed promoter complex (RPc; condition II). However, Gp5.7 had only a very moderate effect on RNAP activity when added after the RPo had formed (condition III, Figure 2D), suggesting that it affects an early step during transcription initiation. Overall, the results clearly demonstrate that Gp5.7 represses *Ec* RNAP activity specifically on T7 early gene promoters during infection; further, it seems that Gp5.7, like Gp2 (7), affects a step *en route* to the RPo.

### Alanine scanning mutagenesis of Gp5.7 reveals functional and structural contributions of each amino acid

The conditional expression of plasmid-borne Gp5.7 in exponentially growing *Ec*, like with Gp2 (12), leads to attenuation of bacterial growth (Supplementary Figure S1A). This implies that Gp5.7 can also potentially repress *Ec* RNAP activity at some host promoters that are essential for growth. We exploited this observation to explore the functional contribution of each amino acid (aa) residue in Gp5.7 by screening a library of Gp5.7 mutants containing an alanine substitution at every position, excluding the starting methionine and seven alanine residues found in the WT Gp5.7 sequence. The results summarised in Supplementary Figure S1B, revealed that, compared to WT Gp5.7, an alanine substitution at six conserved residues (F18, Q19, R24, S35, L42 and W52) markedly (by >90%) reduced the ability of the mutant protein to attenuate *Ec* growth upon induction. Although all the mutant proteins were expressed to at least near WT levels, clear differences in the expression levels were detected between the mutant proteins with respect to the WT protein (Supplementary Figure S1C). Therefore, to investigate whether alanine substitutions at F18, Q19, R24, S35, L42 or W52 adversely affected the structural integrity and stability of the mutant proteins, we performed a comparative analysis of their ^1^H 1D NMR spectra together with that for the WT protein. The amide regions from the NMR spectra of F18A and L42A mutants, shown in Supplementary Figure S1D, exhibit excellent dispersion with several amide resonances at chemical shifts > 8.5 ppm. The data indicate that F18A and L42A mutants are fully folded, while R24A, S35A, Q19A and W52A lack these spectral features suggesting that these mutants possess defects in their structural integrity. Therefore, differences in structural integrity and stability does not seems to account for the differences in the expression levels observed for some of the mutant proteins with respect to the WT protein and we are not able to provide a conclusive explanation for the differences in the expression levels of the mutant proteins. However, of relevance to our analysis, *in vitro* transcription assays revealed that Gp5.7 mutants F18A and L42A failed to detectably repress transcription initiation from the *T7 A1* promoter, suggesting that the reduced RNAP activity at the T7 early gene promoters in the presence of Gp5.7 is specific and that residues F18 and L42 are required for transcription inhibition (Supplementary Figure S1E). However, it is possible that some other residues in Gp5.7 that do not alleviate the attenuation of *Ec* growth may play a role in the specific transcription inhibition of *Ec* RNAP from the *T7 A1, A2* and *A3* promoters.

### Gp5.7 folds into a winged helix-turn-helix (wHTH) like structure

**Figure 3.**
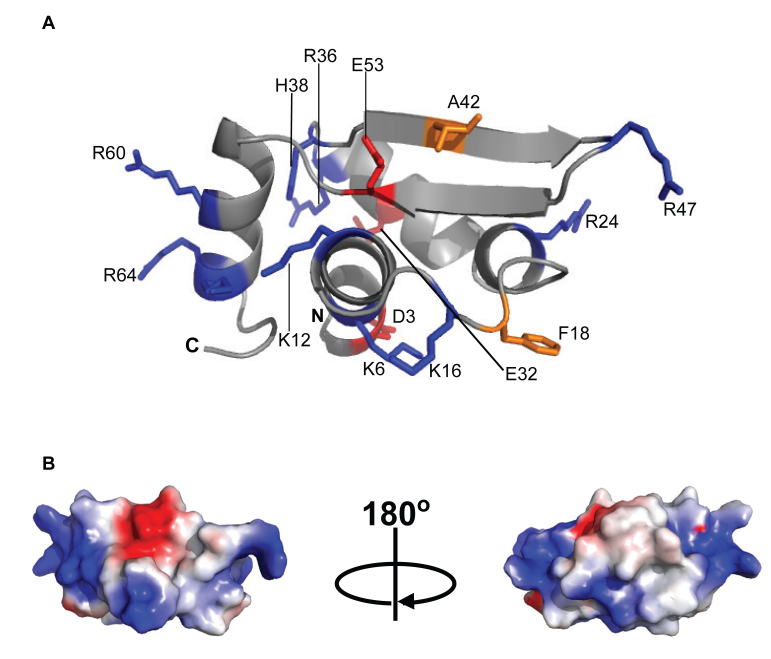
NMR-derived three-dimensional structure of Gp5.7L42A. (**A**) A ribbon representation of Gp5.7L42A indicating the side chains of the positively (blue), negatively (red) charged amino acid residues, mutated residue (L)A42 (orange) and aromatic residue F18 (orange). (**B**) Two views of the molecular surface of Gp5.7L42A (in the same orientation as the ribbon form) colour-coded according to a basic electrostatic surface distribution, calculated using the vacuum electrostatics program in Pymol, version 0.99rc6.

To further understand the repressive effect of Gp5.7 on *Ec* RNAP activity we determined its solution structure using standard multidimensional NMR spectroscopy. Initial evaluation of the NMR spectra for WT Gp5.7 revealed that its quality deteriorated rapidly during the timescale of the experiments (data not shown). Such instability rendered the structure elucidation of WT Gp5.7 intractable. As the L42A mutant protein was significantly more stable and produced a highly structured ^1^H 1D NMR spectrum that is comparable to WT Gp5.7 (Supplementary Figure S1D), we elucidated the solution structure of the Gp5.7L42A (Figure 3A; Supplementary Table S1). The Gp5.7L42A protein folds into a compact globular domain comprising two β strands packed against a four α helical bundle in a α1–α2–α3–β1–β2–α4 arrangement (Figure 3A). A notable feature of the Gp5.7L42A solution structure is that surface exposed charged residues delineate a large positive patch on the α1 and α4 helices (Figure 3A and B). The L(A)42 residue is located on the β1 strand and is surface exposed, consistent with the view that an alanine substitution at this position would not affect the overall conformation of the protein, but could interfere with a functional interaction.

A search for protein structure similarities using the DALI server (18) revealed that the overall structure of Gp5.7L42A exhibits a statistically significant structural similarity to the winged helix–turn–helix (wHTH) motif often found in MarR family of bacterial DNA binding transcription factors (19). The wHTH DNA binding motif of the *Ec* MarR protein (*Z* = 4.6 and an RMSD of 2.9 Å over 61 equivalent Cα atoms) consists of three β strands and α helices with the arrangement of α1–β1–α2–α3–β2–β3, in which the side chains from the three β strands of the ‘wing’ and the three helices enclose the hydrophobic core (Supplementary Figure S2). Similarly, in Gp5.7L42A, the two antiparallel β strands (β1–β2) form the ‘wing’ and the four helices (α1–α4) form the hydrophobic core (Supplementary Figure S2). The loop between α1 and α2 is not well defined in the ensemble of NMR-derived structures and thus likely to be flexible. Gp5.7L42A clearly adopts an atypical wHTH arrangement (α1–α2–α3–β1–β2–α4) (Supplementary Figure S2) with only two β strands composed from 68 aa residues in the wing region, which is significantly shorter than that found in typical wHTH domains (80–100 aa residues).

### Gp5.7 preferentially interacts with the *T7 A1* promoter sequence

**Figure 4.**
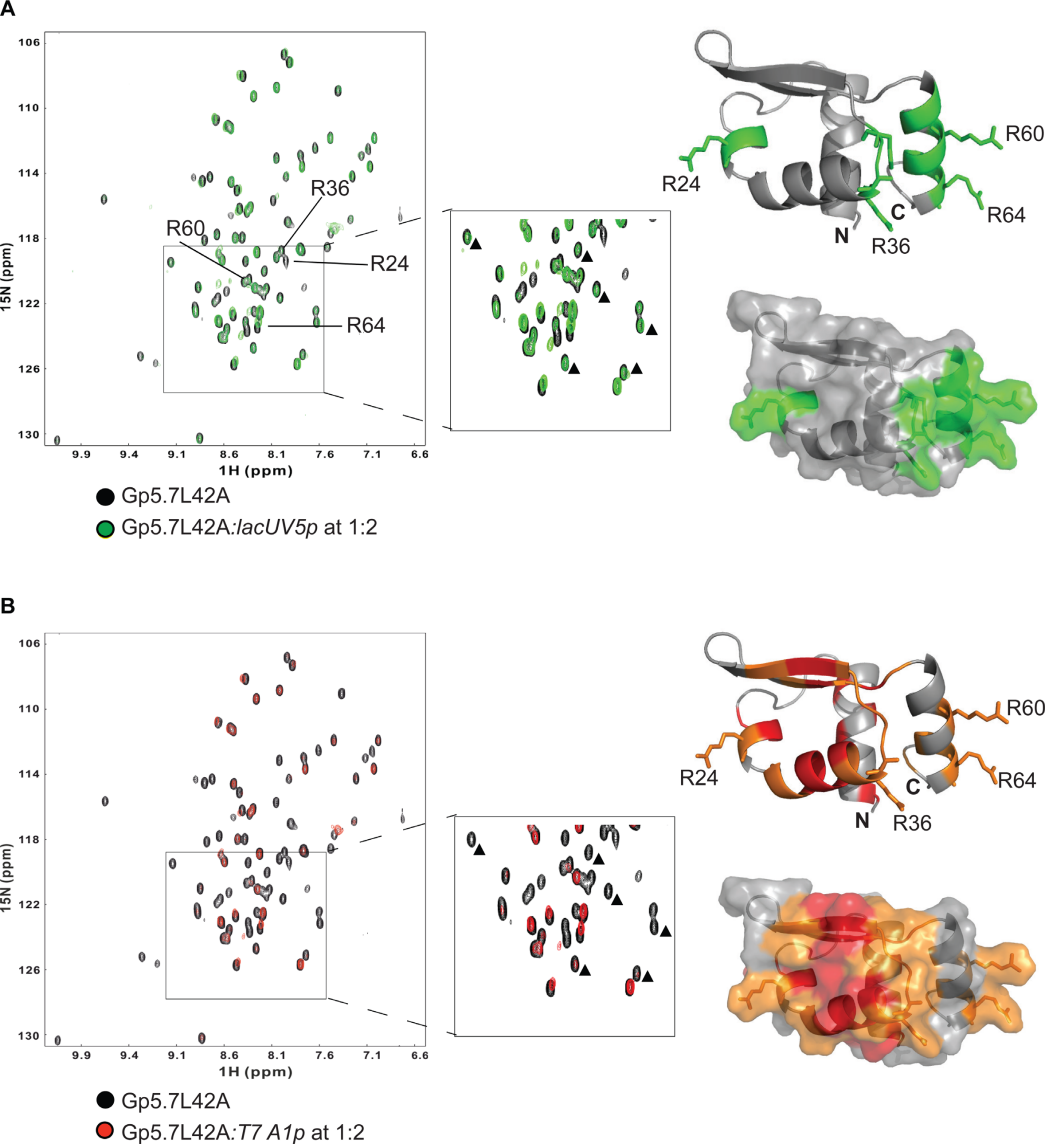
Gp5.7L42A preferentially interacts with the *T7 A1* promoter sequence. (**A**) Overlay of 2D ^1^H–^15^N HSQC spectra of the Gp5.7L42A with and without probe 2 (see Supplementary Table S2) recorded at pH 6.0, 300 K (see key for details). Peaks with moderate broadening are highlighted in green and residues displaying major spectral perturbations are indicated (R24, R36, R60 and R64) in the spectra and in the ribbon and surface representations (right panel) of Gp5.7L42A structure. The residues footnoted by small triangles do not show notable signs of either chemical shift perturbation or peak broadening. (**B**) As in (A) but using probe 1 (see Supplementary Table S2). Red and orange are used to highlight major and moderate, respectively, peak broadening in the 2D ^1^H–^15^N HSQC spectra of the Gp5.7L42A with and without probe 1. The same residues footnoted by small triangles in (A) show significant peak broadening effect.

To determine if Gp5.7 can bind DNA and thus its ability to specifically repress *Ec* RNAP activity at the T7 early gene promoters also could involve an interaction between Gp5.7 and the promoter, we conducted an NMR titration experiment with a ds DNA probe spanning *T7 A1* promoter sequences between –42 and –12 with respect to the transcription start site (at +1) (Supplementary Table S2; probe 1). This sequence was chosen because the binding sites of bacterial MarR protein family members are often located proximal to and/or include the transcription start-site distal consensus promoter element at –35 (19,20). A control DNA probe (probe 2) consisted of the corresponding sequence of the Gp5.7-insensitive *lac*UV5 promoter (Supplementary Table S2; probe 2). We recorded the 2D ^1^H–^15^N HSQC NMR spectra to monitor the backbone amide chemical shift and line-width perturbations in the Gp5.7L42A spectrum in the presence of either DNA probes (∼2-fold molar excess over Gp5.7L42A). Although Gp5.7L42A showed an interaction with both probes, the NMR spectra exhibited two distinct patterns of spectra perturbations depending on which DNA probe was used. In NMR titrations with probe 2 (*lac*UV5 sequence) several peaks exhibited a measurable broadening with the extent of broadening correlating with the amount of probe 2 added (Figure 4A). These residues localised to a positively charged regions located proximal to arginine residues R24, R36, R60 and R64 (Figure 4A). Strikingly, Gp5.7L42A showed a much more pronounced broadening effect at low DNA ratios for probe 1 with a larger number of specific residues affected (*T7 A1* sequence): This broadening effect was observed for residues located together on the β-sheet wing region and α3 (Figure 4B), suggesting higher specificity and affinity for this probe. The peak broadening indicates significant conformation exchange is occurring on an intermediate NMR timescale, the lack of recovery in line-widths at the higher DNA ratios may indicate that an additional state(s) in the bound complex is present. The arginine side chains (R24, R36, R60 and R64), which exhibited perturbations in the presence of probe 2 (*lac*UV5 sequence) are less affected in the presence of probe 1 (*T7 A1* sequence) suggesting that these arginine residues are involved in non-specific, electrostatic interaction with DNA. To localise the specific region bound by Gp5.7L42A within DNA probe 1, we constructed probe 3 (encompassing the sequence between –23 to –12 of *T7 A1* promoter) and probe 4 (encompassing the sequence between –42 to –24 of *T7 A1* promoter) (Supplementary Table S2). As shown in Supplementary Figure S3A, probe 4, but not probe 3, induced a similar pattern of spectral broadening to that for probe 1. We thus truncated probe 4 further and made probe 5 (–40 to –27 of *T7 A1* promoter; Supplementary Table S2), which also induced similar broadening to probe 1 (Supplementary Figure S3A). Some small chemical shift changes can be observed for some residues near the termini, which assuming that these are reporting the same physical process would indicate a *K*_d_ of ∼1 mM for *T7 A1* and at least an order of magnitude weaker for *lac*UV5. To confirm this we made isothermal calorimetry (ITC) measurements for probes 1, 2 and 5. Although we are unable to fully saturate binding in the titration due to limitations in available DNA concentrations, we obtain clear exothermic binding isotherms for *T7 A1* probes 1 and 5, with estimates for the dissociation constants of *K*_d_ of ∼8 and 0.6 ± 0.1 mM, respectively, while binding could not be detected for probe 2 (*lac*UV5) (Supplementary Figure S3B). These data are consistent with NMR observations and overall we conclude that preferential binding exists for Gp5.7L42A to a sequence of the *T7 A1-A3* promoters encompassing the consensus -35 motif (which differs by only one nucleotide: TTGACT in *T7 A1* and TTGACA in *T7 A2* and *A3*). To provide additional evidence that the specific repression of *Ec* RNAP activity by Gp5.7 on *T7 A1-A3* involves an interaction with the region encompassing the consensus –35 motif, we tested whether Gp5.7 affected transcription initiation by Eσ^70^ from the λ *pR* promoter, which has the identical consensus –35 sequence as the *T7 A1* promoter. Results shown in Supplementary Figure S3C clearly indicate that Eσ^70^ activity on the λ *pR* promoter is compromised by Gp5.7 to a similar extent as on *T7 A1* (Supplementary Figure S3C). Overall, we conclude that the specific repression of *Ec* RNAP activity from the *T7 A1-A3* promoter involves an interaction between Gp5.7 and the nucleoprotein complex at the consensus –35 promoter region of these promoters. Although the determined affinity of Gp5.7 to the DNA is weak, it is likely to be enhanced by the interactions Gp5.7 makes with the RNAP in the biologically relevant situation (also see Discussion).

### Gp5.7 is required for optimal T7 growth in *Ec*

**Figure 5.**
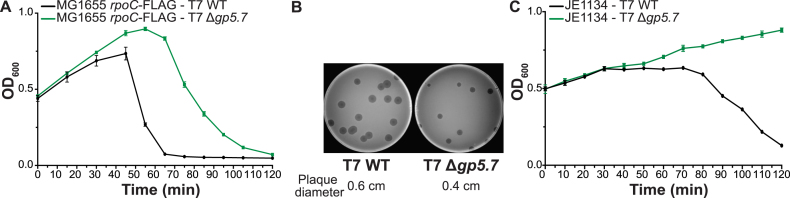
Gp5.7 is required for optimal T7 growth in *Ec*. (**A**) Graph showing the optical density as a function of time of a culture of exponentially growing *Ec* MG1655 *rpoC*-FLAG cells following infection with T7 WT and T7 Δ*gp5.7* phage. (**B**) Plaque morphology of T7 WT and T7 Δ*gp5.7* on *Ec* MG1655 *rpoC*-FLAG. (**C**) As in (A) but using the *Ec* JE1134 strain.

The results indicate that the repression of host RNAP activity by Gp5.7 involves a preferential interaction between the *T7 A1-A3* promoters. We next investigated how the absence of Gp5.7 would affect the ability of T7 phage to grow in *Ec* strain MG1655*rpoC*-FLAG. We initially compared the ability of T7 WT and T7 Δ*gp5.7* to lyse an exponentially growing batch culture of *Ec* by measuring cell density (OD_600_) as a function of time. As shown in Figure 5A, under our conditions, a rapid drop in cell density, indicating cell lysis, was observed after ∼50 min in the *Ec* culture infected with the WT phage. In clear contrast, in the *Ec* culture infected with the T7 Δ*gp5.7* phage, a relatively slower drop in cell density was only observed after ∼70 min. It seems that Gp5.7 is required for optimal growth of T7 in *Ec*. Consistent with this view, the comparison of the plaque morphology of T7 WT and T7 Δ*gp5.7* on *Ec* MG1655 *rpoC*-FLAG revealed that the size of the mutant plaques were smaller (by ∼30%) compared with the T7 WT (Figure 5B). *Ec* strain JE1134 harbours a deletion in the *rpoC* gene that removes aa residues 1149–1190, which comprise the Gp2-binding site (21). Thus, Gp2 does not bind to and inhibit the RNAP in the JE1134 strain, and, as a consequence, T7 does not grow normally in JE1134. However, JE1134 is efficiently infected and lysed by T7 WT, although no progeny phage results (8). Therefore, we used the JE1134 strain as an ‘experimental’ chassis to compare the ability of T7 WT and T7 Δ*gp5.7* to lyse JE1134 cells to determine and provide further evidence that the repression of host RNAP activity by both Gp2 and Gp5.7 is required for optimal development of T7 in *Ec*. Results revealed that, in marked contrast to T7 WT, which lysed the JE1134 cells ∼70 min after infection, the T7 Δ*gp5.7* phage failed to detectably lyse JE1134 cells even after 120 min after infection (Figure 5C). We conclude that an inadequate ability to inhibit the host RNAP significantly compromises the ability of T7 Δ*gp5.7* to develop in *Ec*. Overall, the results indicate that, both Gp5.7 and Gp2, are required for the optimal inhibition of host RNAP and thus growth of T7 in *Ec*.

## DISCUSSION

Despite the wealth of knowledge available on the genetic, molecular and structural basis by which T7 infects *Ec*, as exemplified here, there is much to discover about this simple bacterial virus. The principal finding of this study is that T7 requires, in addition to Gp2, another RNAP inhibiting small protein to temporally control the activity of the host RNAP during infection. Although Gp5.7, unlike Gp2, is dispensable for T7 growth in *Ec*, Gp5.7 is clearly required for optimal infection. Thus, inadequate inhibition of the host RNAP in the absence of Gp5.7 becomes clearly detrimental for T7 development in *Ec* (22). Since phage genomes have evolved to be compact and efficient, why does the T7 phage require three different proteins (Gp0.7, Gp2 and Gp5.7) to achieve one biological outcome? We posit that the genomic location, timing of expression and the dependency of T7 on both host and its own RNAP provides a plausible explanation for the co-existence of Gp0.7, Gp2 and Gp5.7. Infection of *Ec* by T7 begins with the translocation of the left end of the T7 genome into the host which is coupled to the transcription from T7 early genes from *T7 A1-A3* promoters by the *Ec* RNAP. The early gene product, Gp0.7 is a serine/threonine kinase that phosphorylates the *Ec* RNAP at a single site (T1068) in the catalytic β’ subunit (6). Phosphorylation of T1068 residue by Gp0.7 is thought to increase transcription termination at termination sites located between the T7 early (transcribed by the host RNAP) and middle genes (transcribed by the T7 RNAP) (6). Gp2, one of the first middle gene products transcribed by the T7 RNAP and is indispensable for T7 growth in *Ec* (8) binds to a DNA interacting domain of the *Ec* RNAP, called the β’ jaw domain, and inhibits transcription initiation from all three T7 early gene promoters (23). Importantly, however, Gp2 cannot bind to or inhibit actively transcribing RNAP molecules (see later) (7). The beneficial consequences of the action of Gp2 (and Gp0.7) for the T7 phage would be to decrease host RNAP read-through into middle genes in order to avoid the slowly transcribing host RNAP interfering with the fast-transcribing T7 RNAP. During normal T7 DNA replication and packaging, the T7 RNAP recognises the genomic end of each separate unit of the progeny T7 genomes that are organised as concatemers, presumably by pausing at a unique site located immediately after the concatemeric junctions. The pausing T7 RNAP is likely to serve as a signal to recruit DNA packaging factors Gp18 and Gp19. A model proposed by Qimron *et al*. (22), envisages that, in the absence of Gp2, continuous transcription from the strong host RNAP-dependent early gene promoters (*T7 A1-A3*) during the infection cycle, could create a ‘roadblock’ to transcription by the much faster transcribing T7 RNAP leading to undesired pausing of the T7 RNAP. This, in turn, could result in the untimely aberrant recruitment of DNA packaging factors and production of less than unit length phage genomes leading to an unsuccessful infection of *Ec* by T7 phage. In support of this view, T7 infection in the absence of Gp2 fails not at the stage when the switch from host to T7 RNAP occurs, but much later, during T7 DNA packaging (24).

During the course of the infection, two middle gene products, Gp3 and Gp6, which are exo- and endonucleases, respectively, degrade the host chromosome and thereby provide resources to drive phage gene expression and DNA replication. However, the degradation of host chromosome also results in the release of host chromosome-bound proteins, such as actively transcribing (therefore Gp2 unbound; see above) host RNAP molecules or HNS (25), for which now the progeny T7 genome becomes the only available target in the cell. Aberrant binding of HNS to or host RNAP activity on the progeny T7 genomes would be detrimental. Gp5.5 counteracts the inhibitory effect of *Ec* HNS on phage DNA gene expression (10,26). Since the strong host RNAP-dependent early gene promoters *T7 A1-A3* are the only target for the host RNAP following host chromosome degradation by Gp3 and Gp6 (which would further explain the weak affinity of Gp5.7 to DNA as it does not have to compete for DNA sites), our results indicate that Gp5.7 could provide another layer of control to specifically counteract the host RNAP activity at *T7 A1-A3* that has escaped inhibition of Gp2 at this point in the infection cycle to ensure optimal infection outcome. Since Gp5.7 interacts with *both* the RNAP (Figure 1) and DNA (Figure 4 and Supplementary Figure S3B), we envisage a scenario where the mechanism of inhibition of transcription initiation does not simply involve the occlusion of RNAP from the *T7 A1-A3* promoters, but could involve the binding of Gp5.7 to the RNAP first (since Gp5.7 seems to have a higher affinity to the RNAP than to DNA) thereby preventing productive (but not initial) promoter complex formation on the *T7 A1-A3* promoters. Although deciphering the precise binding site of Gp5.7 on *Ec* RNAP and the mechanistic basis by which Gp5.7 represses *Ec* RNAP activity will be the subject of future research, the results of this study underscore that the bacterial RNAP is not only a nexus for the regulation of bacterial gene expression, but also represents an essential target for the diversity of mechanisms by which different phages acquire and modulate the activity of their host transcription machinery to ensure optimal infection outcome.

## Supplementary Material

Supplementary Data
