# Supplementary material for: Full shut-off of Escherichia coli RNA-polymerase by T7 phage requires a small phage-encoded DNA-binding protein
Source: Nucleic Acids Res. 2017 May 9;45(13):7697–707. doi: 10.1093/nar/gkx370 (PMC5569994; doi:10.1093/nar/gkx370)
Supplement: Supplementary Data [file gkx370_supp.pdf]

SUPPLEMENTARY DATA

SUPPLEMENTARY FIGURE LEGENDS

Figure S1. Alanine scanning mutagenesis of Gp5.7. **(A)** Induction of plasmid-borne Gp5.7 expression in exponentially growing *Ec* results in attenuation of growth. Black arrow indicates t=0 when Gp2 (positive control) or Gp5.7 expression was induced by the addition of L-arabinose. **(B)** Activity of an alanine mutant library of Gp5.7 in the context of the growth inhibition assay shown in (A). The fold-change in the OD_{600} value for each mutant relative to WT 4 hours after Gp5.7 expression is plotted in the bar chart. The alignment of protein sequences of Gp5.7 homologues from other phages indicates that an alanine substitution at six conserved residues (F18, Q19, R24, S35, L42 and W52) markedly reduced the ability of the mutant protein to attenuate *Ec* growth. **(C)** Image of a representative Western blot containing whole-cell lysates of *Ec* containing the six mutants that failed to attenuate *Ec* growth probed with anti-His (to detect Gp5.7) and anti-α subunit of the *Ec* RNAP (as a loading control). The values calculated for the signal intensity represent values from at least two independent experiments and fall within 10% of the value shown. **(D)** The ^{1}H 1D NMR spectra of the six mutants that failed to attenuate *Ec* growth compared to that of WT protein. Mutant proteins (L42A and F18A) that retain structural integrity comparable to that of the WT proteins are shown on the left; mutant proteins (Q19A, R42A, S35A and W52A), which retain only parts of their structural features, are shown on the right. **(E)** Autoradiograph of an *in vitro* transcription assay demonstrating that the F18A and L42A Gp5.7 are markedly compromised to repress *Ec* RNAP activity at the *T7 A1* promoter. The percentage of RNA transcript synthesised (%A) in the reactions with Gp5.7 with respect to reactions with no Gp5.7 is given at the bottom of the gel and the value obtained in at least three independent

experiments fell within 3-5% of the %A value shown. The dinucleotide used in the assay is underlined and the asterisks indicate the radiolabelled nucleotide(s).

Figure S2. Gp5.7 folds into a winged helix-turn-helix (wHTH) like structure. **(A)** Comparison of the structures of Gp5.7L42A and the wHTH DNA binding domain of *Ec* MarR protein. *Left panel*. Topology of Gp5.7L42A. *Middle* and *right* panels. Cartoon representation and topology of the wHTH DNA binding domain of *Ec* MarR protein (PDB ID: 5LGM). **(B)** Sequence alignment of Gp5.7 with MarR protein: '|' represents identical residues, ':' indicates highly conserved residues and '.' shows a lower degree of conservation.

Figure S3. Gp5.7 preferentially interacts with the *T7 A1* promoter sequence. **(A)** As in Figure 4 but the NMR titration experiments were done with probes 3, 4 and 5 (see Table S2 and text for details) and Gp5.7L42A. **(B)** Isothermal Titration Calorimetry (ITC) titration profile of Gp5.7L42A with DNA probes 1, 2 and 5. In the upper panels, the baseline corrected experimental data for Gp5.7L42A is shown; in the lower panels, molar heats of binding are plotted against the protein to ds DNA molar ratio. Fitting of ITC data (shown as solid line) was done using model for one set of binding given in the software (See Materials and Methods). **(C)** Autoradiograph of denaturing gels showing the ability of the *Ec* RNAP holoenzyme to synthesise a dinucleotide-primed RNA product from the λ *pR* promoter. The dinucleotide used in the assay is underlined and the asterisks indicate the radiolabelled nucleotide(s).

SUPPLEMENTARY TABLES

Table S1. Structural statistics from the solution structure calculation for Gp5.7L42A (PDB ID: 5LGM)

**NMR Distance and Dihedral Constraints**	
Distance constraints	
Total NOE	875
Intraresidue	354
Interresidue	521
Sequential (|*i-j*|)=1)	181
Short range (2≤|*i-j*|)≤3)	87
Medium range (4≤|*i-j*|)≤5)	56
Long range (|*i-j*|)>5)	197
Total Dihedral angle Restraints	108
Φ	54
Ψ	54
Total RDCs	0
**Structural Statistics**	
Violations (mean and SD)	
Distance constraints (Å)	0.024 ± 0.007
Dihedral angle constraints (°)	0.30 ± 0.073
Maximum dihedral angle violation (°)	0.64
Maximum distance constraint violation (Å)	0.22
Deviations from idealized geometry	
Bond length (Å)	0.0015 ± 0.000
Bond angle (°)	0.319 ± 0.006
Impropers (°)	0.263 ± 0.011
Average Pairwise rmsd$^{\alpha}$(Å)	
Heavy	0.576 ± 0.0843
Backbone	0.216 ± 0.0470

Table S2. Promoter probes used in the chemical shift experiments

Probes	DNA name	Sequence
**1**	*T7 A1p* (-42 to -12)	AGAGTA**TTGAC**TTAAAGTCTAACCTATAG**GA**<br>TCTCAT**AACTG**AATTTCAGATTGGATATC**CT**
**2**	*lacUV5p* (-42 to -12)	CCAGGC**TTTACA**CTTTATGCTTCCGGCTCG**T**<br>GGTCCG**AAATGT**GAAATACGAAGGCCGAGC**A**
**3**	*T7 A1p* (-23 to -12)	TAACCTATAG**GA**<br>ATTGGATATC**CT**
**4**	*T7 A1p* (-42 to -24)	AGAGTA**TTGAC**TTAAAGTC<br>TCTCAT**AACTG**AATTTCAG
**5**	*T7 A1p* (-40 to -27)	AGTA**TTGAC**TTAAA<br>TCAT**AACTG**AATTT

*-35 and -10 elements are in bold and underlined.

Table S3. Sequences of primers and oligonucleotides used to generate the promoter probes in this study

Primers	Sequence (5' to 3')
AS1	TTACATATGTCTGACTACCTGAAAGTGCTGC
AS2	TTTGGATCCTTAGACACAACCTCCCATTCGT
T7p_tR2_1	TAATACGACTCACTATAGG
T7p_tR2_2	GTAAAACGACGGCCAG
MG5F	GTACCATATGGATAAGGACTCTTTCAAACAATCTCCTGCAACAGTACGGGAGGTGTTCTGTTACGCCAGGT TAGCGTCGA
MG5R	TAAGTCTAGACTGTATATCGTTCCACACATCGCGTGGAATAGTCACAAGGTCACGAGACATTACGCCAGGT TAGCGTCGA
**Promoter Probes***	
*lacUV5p*_NT (-45/+20)	ACCCCAGGC**TTTACA**CTTTATGCTTCCGGCTCG**TATAAT**GTGTGG**A**ATTGTGAGCGGATAACAATT
*lacUV5p*_T (-45/+20)	AATTGTTATCCGCTCACAATTCCACACATTATACGAGCCGGAAGCATAAAGTGTAAAGCCTGGGGT
*N25p*_NT (-45/+20)	AAAAATTTAT**TTGCTT**TCAGGAAAATTTTTCTG**TATAAT**AGATTC**A**TAAATTTGAGAGAGGAGTT
*N25p*_T (-45/+20)	AACTCCTCTCTCAAATTTATGAATCTATTATACAGAAAAATTTTCCTGAAAGCAAATAAATTTTT
*galP1p*_NT (-45/+20)	TTCCATGTCACACTTTTCGCATCTTTGTTA**TG**C**TATGGT**TATTTC**A**TACCATAAGCCTAATGGAG
*galP1p*_T (-45/+20)	CTCCATTAGGCTTATGGTATGAAATAACCATAGCATAACAAAGATGCGAAAAGTGTGACATGGAA
*T7 A1p*_NT (-45/+20)	AAAAGAGTA**TTGACT**TAAAGTCTAACCTATAG**GATACT**TACAGCC**A**TCGAGAGGGACACGGCGAA
*T7 A1p*_T (-45/+20)	TTCGCCGTGTCCCTCTCGATGGCTGTAAGTATCCTATAGGTTAGACTTTAAGTCAATACTCTTTT
*T7 A2p*_NT (-45/+14)	AAACAGGTA**TTGACA**ACATGAAGTAACATGCAG**TAAGAT**ACAAATC**G**CTAGGTAACACTA
*T7 A2p*_T (-45/+14)	TAGTGTTACCTAGCGATTTGTATCTTACTGCATGTTACTTCATGTTGTCAATACCTGTTT
*T7 A3p*_NT (-45/+14)	AACAAAACGG**TTGACA**ACATGAAGTAAACACGG**TACGAT**GTACCAC**A**TGAAACGACAGTG
*λ pRp*_NT (-45/+20)	CCGTGCGTG**TTGACT**ATTTTACCTCTGGCGGT**GATAAT**GGTTGC**A**TGTACTAAGGAGGTTGTAT
*λ pRp*_T (-45/+20)	ATACAACCTCCTTAGTACATGCAACCATTATCACCGCCAGAGGTAAAATAGTCAACACGCACGG

*Shown in bold are the -35 and -10 consensus promoter elements on the non-template strand of each promoter used in this study; the transcription start site is underlined and shown in bold.

SUPPLEMENTARY FIGURES

Figure S1.

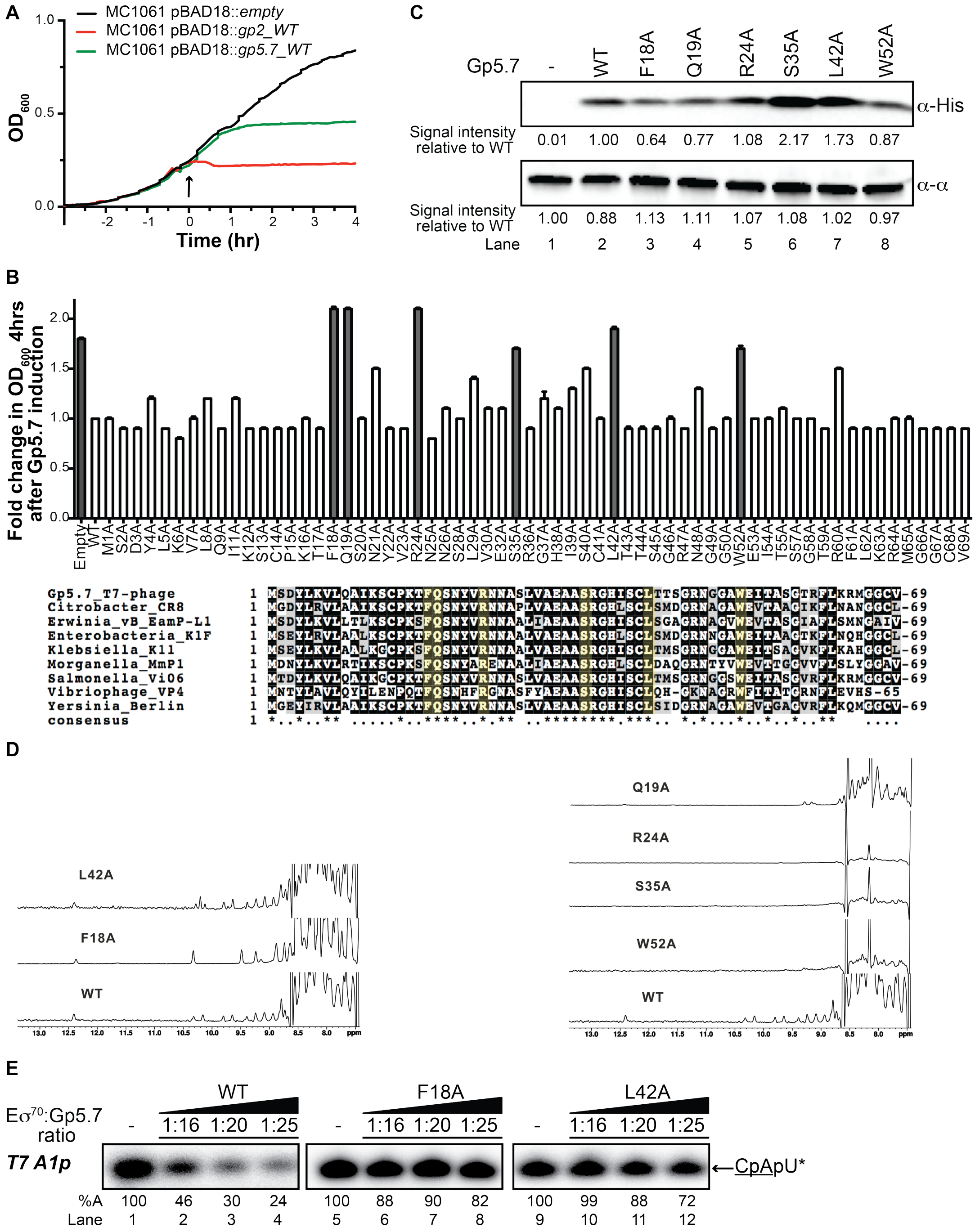

Figure S2.

A

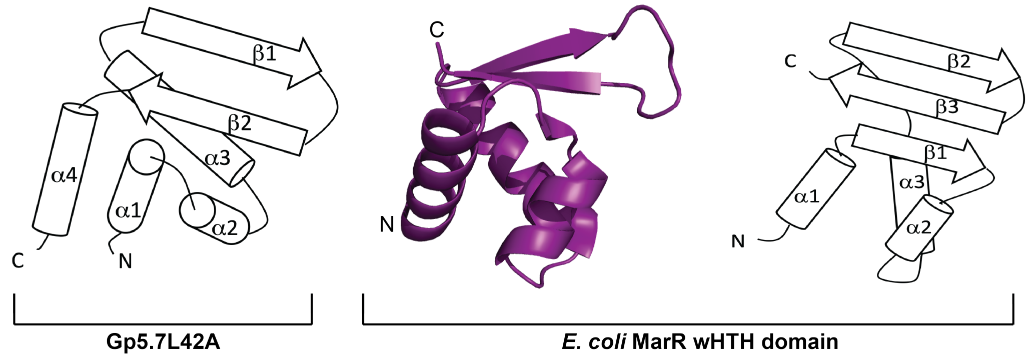

B

```
Gp5.7      1 ----------------------------------------MSDYLKVLQA     10
                                                     ...|:.:| :
MarR       1 GMDYSNELKELFLMNQTYATLFTLTNKIQIEGDKYFGILTSRQYMTIL-S     49

Gp5.7     11 IKSCP--KTFQSNYVR-------NNASLVAEAASRGHISCL-TTSGRNGG     50
             |...|  :|..:|..|       |...|||.....|::..: :...:...
MarR      50 ILHLPEEETTLNNIARKMGTSKQNINRLVANLEKNGYVDVIPSPHDKRAI     99

Gp5.7     51 AWEITASGTRFLKRMGGCV-------------------------------     69
             ..::|..|.   |.|..|.
MarR     100 NVKVTDLGK---KVMVTCSRTGINFMADVFHEFTKDELETLWSLLKKMYR    146

Gp5.7     70 -------------------------------------------     69

MarR     147 FNGEEQDGFEEDANFMEYEEIDKIKSEALEEFAKRRNRVNKND     189
```

Figure S3.

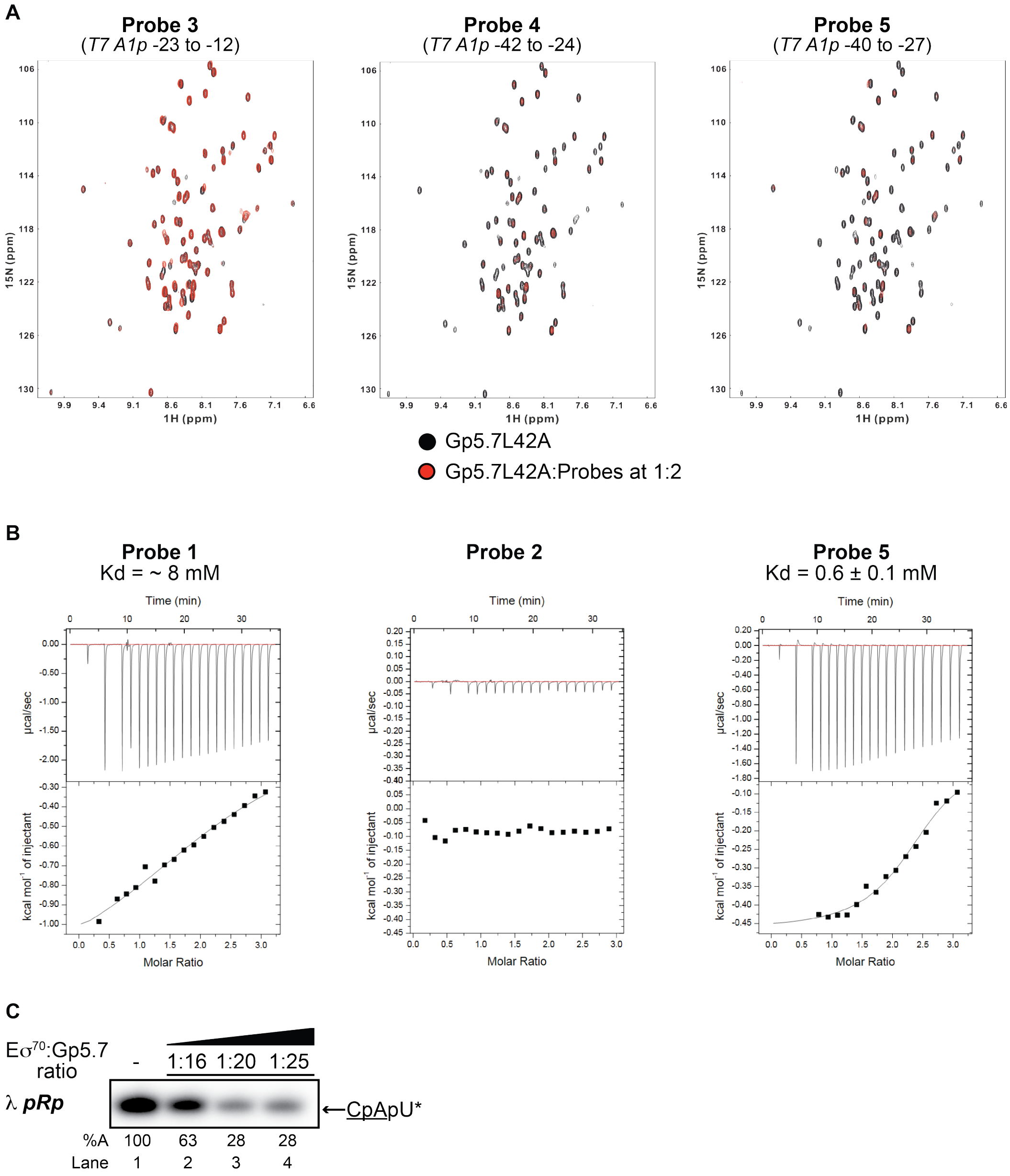
A
Probe 3
(T7 A1p -23 to -12)
Probe 4
(T7 A1p -42 to -24)
Probe 5
(T7 A1p -40 to -27)
15N (ppm)
1H (ppm)
Gp5.7L42A
Gp5.7L42A:Probes at 1:2
B
Probe 1
Kd = ~ 8 mM
Probe 2
Probe 5
Kd = 0.6 ± 0.1 mM
Time (min)
µcal/sec
kcal mol-1 of injectant
Molar Ratio
C
Eσ70:Gp5.7
ratio
1:16 1:20 1:25
λ pRp
←CpApU*
%A 100 63 28 28
Lane 1 2 3 4
